# Annotation of protein residues based on a literature analysis: cross-validation against UniProtKb

**DOI:** 10.1186/1471-2105-10-S8-S4

**Published:** 2009-08-27

**Authors:** Kevin Nagel, Antonio Jimeno-Yepes, Dietrich Rebholz-Schuhmann

**Affiliations:** 1 European Bioinformatics Institute, Wellcome Trust Genome Campus, Hinxton, Cambridge, CB10 1SD, UK

## Abstract

**Background:**

A protein annotation database, such as the Universal Protein Resource knowledge base (UniProtKb), is a valuable resource for the validation and interpretation of predicted 3D structure patterns in proteins. Existing studies have focussed on point mutation extraction methods from biomedical literature which can be used to support the time consuming work of manual database curation. However, these methods were limited to point mutation extraction and do not extract features for the annotation of proteins at the residue level.

**Results:**

This work introduces a system that identifies protein residues in MEDLINE abstracts and annotates them with features extracted from the context written in the surrounding text. MEDLINE abstract texts have been processed to identify protein mentions in combination with taxonomic species and protein residues (F1-measure 0.52). The identified protein-species-residue triplets have been validated and benchmarked against reference data resources (UniProtKb, average F1-measure of 0.54). Then, contextual features were extracted through shallow and deep parsing and the features have been classified into predefined categories (F1-measure ranges from 0.15 to 0.67). Furthermore, the feature sets have been aligned with annotation types in UniProtKb to assess the relevance of the annotations for ongoing curation projects. Altogether, the annotations have been assessed automatically and manually against reference data resources.

**Conclusion:**

This work proposes a solution for the automatic extraction of functional annotation for protein residues from biomedical articles. The presented approach is an extension to other existing systems in that a wider range of residue entities are considered and that features of residues are extracted as annotations.

## Introduction

The understanding of the biological function of proteins remains to be a central challenge in biology. In protein science, sequence analysis of amino acids or studies of their spatial distribution have led to predictions and discoveries of a number of biological significant patterns and motifs, e.g. metal-binding sites, catalytic triads, and ligand binding sites [1-7]. Complementary to these mined data is the proliferation of protein annotations by extracting information from biomedical articles in the view of updating existing databases. For example, scientific articles have been used to generate the specialised databases DOLOP (lipoprotein) [8], and TMPDB (transmembrane proteins) [9]. Clearly, annotations can be used to verify data mined sequence or structure patterns and likewise an identified pattern provides a context to interpret functional annotations. However, major annotation efforts are currently focused on the identification of features for whole proteins whereas the annotation of protein residues is left behind. The annotation of protein residues is vital for the biological community because the information either points to or it implies a functional site in a protein. This is also reflected in the field of automatic information extraction from literature, where solutions have been published for the extraction of interactions of proteins [10,11], subcellular protein localisation [12], pathway discovery [13], and function annotation with Gene Ontology terms [14]. Few groups have investigated in point mutation extraction, but so far without feature extraction for residue annotation [15-19].

Works have been published in the biomedical text mining community that focused on the extraction of point mutations, which is one type of a residue mention but others have to be considered as well [15-19]. The point mutation extraction systems called MEMA [18] and MuteXt [19] use a dictionary lookup approach to detect protein names and disambiguate multiple protein-residue pairs with a word distance measurement. MutationGraB [15], the successor of MuteXt, uses a graph bi-gram method to calculate the proximity by weighting the association of word-pairs. Two other applications called MutationMiner [16,20] and mSTRAP [21] focus on the integration of extracted point mutations into a protein structure visualisation program. A recently published extraction system finds sequence variants in MEDLINE to assist manual curation efforts for single amino acid polymorphisms of human proteins [22]

All of the listed text mining systems are dedicated to the extraction of point mutations, but do not extract contextual features from text as functional annotations of protein residues. A recent publication [16] proposes an ontological model that should hold information extracted from MutationMiner as well as point mutation annotations. The authors did not investigate into feature extraction methods. Residue annotation differs from functional annotation of proteins because the biological role of a residue is described in a biochemical context, which is then revealed in the function or property of the protein. At present, there is neither such an ontological model nor a terminological resource publicly available. The goal of our research is the identification of biological functions linked to protein structure patterns that have been identified in a data mining approach applied to Protein Data Bank (PDB). This paper reports on the text mining solution for this combined mining approach and on the compilation of protein residue annotations that support interpretation of structure patterns. The result demonstrates that textual evidence linked to protein residue mentions in text can be used to contribute to the annotations in UniProtKb. Our analysis identifies and categorizes contextual features from MEDLINE abstract texts that contribute to the annotation of protein structures with respect to their biological function.

The contribution of this work is the automatic extraction of protein residue annotation from biomedical articles. Contextual information are exploited to identify features of residues that correspond to one of six chosen target categories. As a result, proteins can be selected with residues clustered by annotation types, which can lead to, for example, the identification of active site residues.

## Methods

**Figure 1:**
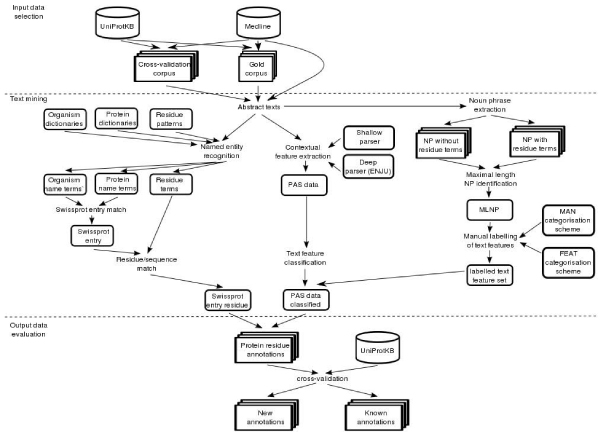
**Overview of text mining processes and evaluation methods for the extraction of functional annotation**. The presented functional annotation extraction system consists of two major text mining processes: protein residue identification (left hand side), and contextual feature extraction (right hand side). The extracted annotations are compared with information in the feature table from UniProtKb.

The extraction of functional annotations for protein residues from literature consists of two parts: protein residue identification, and contextual feature extraction. Figure 1 illustrates the procedures of the developed information extraction system.

### Protein residue identification

Named entity recognition for proteins was based on a combination of dictionary lookup with fuzzy matching and basic disambiguation [23-25]. All protein names were collected from UniProtKb/SwissProt [26]. Similarly, names of species were extracted from the NCBI Taxonomy [27]. The dictionary was complemented with terminologies describing only the referenced genus and the collection of full organism name (genus + species) augmented with abbreviated genus forms (first letter abbreviation of genus + species). Web services for the identification of protein names and taxa names are available from the Text Mining infrastructure at the EBI [23].

The extraction of residue mentions follows approaches of previous publications by reusing a selection of regular expression patterns for point mutation [18,19]. The set of expression patterns was extended to identify three types of residue mentions: wild-type forms, mutation mentions, and range or pair of residues (cf. table 1). The first basic type is the single protein sequence site reference which consists of a (wild-type) amino acid name followed by the sequence position number, e.g. "Gly-12", "arginine 4", "Tyr74", "Arg(53)". A point mutation is described by the change of an amino acid at a given position, e.g. "W77R", "Cys560Arg", "ser-52->ala", "ala2-methionine". Finally, the third type of residue site describes either a list of residues or an interaction pair, e.g. "Tyr 85 to Ser 85", "Trp27-Cys29". The common notation is an amino acid name, sequence position, a connection symbol or connection word, amino acid name, and sequence position. In addition, we have also developed other patterns to cover grammatical expressions of residue mentions, such as "isoleucine at position 3", "substitution of Ala at position 4 to Gly", "Ser472 to glutamic acid".

**Table 1:** Regular expression patterns for the detection of residue mentions in text. The patterns recognise single (SITE) or multiple wild-type residue sites (SITES), a sequence range or residue pair (RANGE/PAIR), and point mutation (MUTATION). The set covers abbreviated notations of residues as well as grammatic expressions found in text.

RANGE-TO	= ("-"+ ("to" "-+")? |"to");
CONVERT-TO	= ("to"|"-"+">"?);
XAA	= ("X"|"XAA"|"xaa");
POS	= (1–9)(0–9)*;
RESN1	= [ARNDCQEGHILKMFPSTWYVOUBZX];
RESN3	= ([aA]la|ALA | [aA]rg|ARG | [aA]sn|ASN | [aA]sp|ASP | [cC]ys|CYS
	| [gG]ln|GLN | [gG]lu|GLU | [gG]ly|GLY | [hH]is|HIS | [iI]le|ILE
	| [lL]eu|LEU | [lL]ys|LYS | [mM]et|MET | [pP]he|PHE | [pP]ro|PRO
	| [sS]er|SER | [tT]hr|THR | [tT]rp|TRP | [tT]yr|TYR | [vV]al|VAL
	| [pP]yl|PYL | [sS]ec|SEC | [aA]sx|ASX | [gG]lx|GLX | [xX]aa|XAA);
RESNF	= ([aA]lanine | [aA]rginine | [aA]sparagine | [aA]spart(ate|ic acid) | [cC]ysteine
	| [gG]lutamine | [gG]lutam(ate|ic acid) | [gG]lycine | [hH]istidine | [iI]soleucine
	| [lL]eucine | [lL]ysine | [mM]ethionine | [pP]henylalanine | [pP]roline
	| [sS]erine | [tT]hreonine | [tT]ryptophan | [tT]yrosine | [vV]aline
	| [pP]yrrolysine | [sS]elenocysteine | [aA]spartic acid or [aA]sparagine
	| [gG]lutamic acid or [gG]lutamine);
SITE	= ((RESN3 | RESNF) POS "residue"?
	| (RESN3 | RESNF)"-"+ POS "residue"?
	| (RESN3 | RESNF)"residue"? "at position"? POS "residue"?
	| (RESN3 | RESNF)"("POS")" "residue"?
	|"amino acid"? "residue" "at position"? POS
	|"amino acid" "residue"? "at position"? POS
	| RESNF "residue" POS);
SITES	= (RESNF"s"((","|"and"|"or") RESNF"s")*
	| RESNF"s"? ("at position" "s"?)? (","|"and"|"or") (("at position" "s"?)? (","|"and
	|"or") POS)+
	| RESNF "residue" "s"?
	| RESN3 "residue" "s"? ("at position" "s"?)? POS (("at position" "s"?)? (","|"and" | "or") POS)+

	| RESN3 "residue" "s"?
	|"residue" "s"? ("at position" "s"?)? POS (","|"and"|"or") POS)+
	| (RESN3 | RESNF)"for"(RESN3 | RESNF)"at position"POS (","|"and"|"or") POS)+
	| RESNF ("," | "and" | "or") POS)* "residue" "s"?);
RANGE/PAIR	= ("residue" "s"? ("," | "and" | "or") RANGE-TO POS)+
	|"amino acid" "residue"? "s"? ("," | "and" | "or") RANGE-TO POS)+
	| ("resiude" "s"?)? "at position" "s"? ("," | "and" | "or") RANGE-TO POS)+
	| RESI RANGE-TO RESI);
MUTATION	= (RESN1 POS RESN1
	| RESN1 "-" POS "-" RESN1
	| RESN1 "(" POS ")" RESN1
	| RESI CONVERT-TO (RESN3 | RESNF)
	| RESI RESN3
	|"from" (RESNF | RESN3) CONVERT-TO (RESNF | RESN3)"at position"POS
	| (RESN3 | RESNF) "for" (RESN3 | RESNF) "at position" POS
	| RESI ("-"+ | CONVERT-TO) RESI "substitution");

The identification of the entity triplet organism, protein, and residue, is based on the algorithm described by [19] with some modifications. Although the association between protein and residue can also be done in absence of organism detection, e.g. by assuming one of the most popular model organisms, or by inference of a detected disease name in the text, we did not follow this approach as this could lower the precision of the identification system.

In the first step proteins were associated with their hosting organisms. All pairs of protein-species were determined for each protein in a text and ranked by the smallest word distance between the entities in the pair. The identification of organism-protein began with the pair with the smallest word distance measure. A valid association was found, if the relation was specified in UniProtKb. If an association was validated then the search was terminated, and the protein was annotated with the corresponding Uniprot identifier, otherwise the next entity pair from the list was tested. If no match between protein and species was found, then the search was relaxed to genus matching. This relaxed matching is the expansion to the [19] algorithm. Because entries in UniProtKb are species specific, the protein-genus association will result in a list of Uniprot identifiers as annotation of the protein.

The second step of entity triplet association was the association of residues with their source proteins. The procedure of selecting and ranking the residue-protein pairs was similar to the protein-organism association identification. For each pair that was to be tested the annotated Uniprot identifier of the protein was used to retrieve the protein sequence from the database. Three cases can be distinguished: (1) the residue correctly matches the protein sequence; (2) several alternative sequences are matching from a list of proteins; and (3) no match can be found for the residue with the available protein sequences. If a match was not found, then the protein-residue pair was rejected, and the search continued with the next pair from the ranked list. Notice that protein-residue association is purely based on the identification of a residue in a protein sequence. A grounding step was not utilised as the identification of the correct sequence position of a residue would require either the simultaneous alignment of residues, or the use of a commonly agreed and standardised sequence indexing scheme.

### Feature extraction for the annotation of residues

The extraction of contextual features of protein residue mentions is based on a syntactical analysis of natural language sentences. Two approaches were developed in this work and compared in the performance evaluation study: shallow parser based relation extraction, and full parser based relation extraction.

The first approach was to develop a shallow parser, which aims to find the boundaries of major constituents in a sentence, such as noun phrases. The design is based on heuristics and the idea of finding general relations between closed-class English words [28]. All prepositional and verbal relations between noun phrases are extracted from text.

Initially, an abstract text was split into sentences, and then annotated with part-of-speech (POS) tags using the CISTAGGER [29], which was trained on biomedical texts. Based on a rule set and the POS information the developed shallow parser identifies noun phrases, verb groups, verb phrases, and prepositional phrases for an analysed sentence (cf. table 2). Based on the determined phrase structure, the parser then extracts verbal relations of noun phrases or prepositional phrases. A condition of the extraction is, that at least one relation element must contain one or more residue mentions. The extracted relation is then transformed to fill the slots of a predicate-argument structure (PAS) [30].

**Table 2:** Rule set for shallow parsing. The rules are used to identify general verbal and prepositional relations between noun phrases in text. N is a noun, Det a determiner, Adj an adjective, Adv an adverb, P a preposition, NP a noun phrase, PP a prepositional phrase, VP a verb phrase, VG a verb group, and REL is the target relation. Notice, that the grammar does not consider coordinating conjunctions, e.g. with "and", "or" and ",".

NP = Det? (Adj|Adv|N)* N
PP = P NP
VG = (Adv|Aux|V|InfTo)* V
VP = VG NP PP*
REL = NP PP* VP

The second approach in contextual feature extraction utilises the full parser ENJU [31] (version 2.3), which generates a head-driven parse tree from a sentence. Because the output contains a lot of information, different interpretations of the parse structure are possible. In this study, a wrapper was developed that converts the parser's output into the presented PAS data format. The assumption is that by following the direct links of a verb to its arguments in the tree, and then collecting all the sub-branches of each argument, the phrase structure of a verb argument can be found. The identified NP PP* VP structures are then decomposed to fill the PAS template.

### Categorisation of contextual features

The semantic interpretation of contextual features, which are the arguments of the extracted PAS, relies on the endogenous classification approach described by [32]. During the training phase, lexical constituents of multi-word terms were extracted from a reference set.

They represent the features of the predefined categories. These terms were manually labeled according to a categorisation scheme (cf. table 3). To identify relevant terms that co-occur with residue mentions in MEDLINE we used maximal length noun phrase analysis [33] (cf. Additional file 1).

**Table 3:** Biological catagories for the interpretation of functional annotations. The interpretation of extracted annotations is based on the automatic assignment of semantic labels to the arguments of a PAS. Because a comprehensive ontology is not available two categorisation schema are tested in this study. The first is the design of a scheme (MAN) based on an analysis of relevant MEDLINE sentences for residue annotation (bottom-up approach). Alternatively, the categories in the feature table of UniProtKb (FEAT) can be reused (top-down approach). Both categorisation schemes reflect concepts of biological interest. However the bottom-up approach has the advantage that proposed categories are data-driven, while in a top-down approach examples of listed categories may not be present in natural language text, or other categories are missing in the scheme.

MAN		FEAT	
	
Category	Defintion	Category	Defintion
STR_COMP	Structure component. Class denoting concepts that represent pieces and parts of the protein structure.	DOMAIN	Extent of a domain, which is defined as a specific combination of secondary structures organised into a characteristic three-dimensional structure of fold.
		
		MOTIF	Short (up to 20 amino acids) sequence motif of biological interest.
		
		TOPO_DOM	Topological domain.
		
		CHAIN	Extent of a polypeptide chain in the mature protein.
		
		TRANSMEM	Extent of a transmembrane region.
		
		COILED	Extent of a coiled-coil region.

CHEM_MOD	Chemical modification. Class denoting changes to the protein sequence and the chemical composition.	VARIANT	Authors report that sequence variants exist.
		
		MOD_RES	Posttranslational modification of a residue.
		
		PEPTIDE	Extent of a released active peptide.
		
		VAR_SEQ	Description of sequence variants produced by alternative splicing, alternative promoter usage, alternative initiation and ribosomal frameshifting.
		
		LIPID	Covalent binding of a lipid moiety.
		
		CARBOHYD	Glycosylation site.

STR_MOD	Structural modification. Class denoting the changes to the protein structure without changes to the chemical composition.	REGION	Extent of a region of interest in the sequence.
		
		SITE	Any interesting single amino-acid site on the sequence, that is not defined by another feature key.

BINDING	Binding type. Class denoting different physico-chemical forces leading to a bond formation between a protein structure component and a chemical entity.	BINDING	Binding site for any chemical group (co-enzyme, prosthetic group, etc.).
		
		METAL	Binding site for a metal ion.
		
		DISULFID	Disulfide bond.
		
		CROSSLNK	Posttranslationally formed amino acid bonds.
		
		DNA_BIND	Extent of a DNA-binding region.
		
		NP_BIND	Extent of a nucleotide phosphate-binding region.
		
		ZN_FING	Extent of a zinc finger region.
		
		CA_BIND	Extent of a calcium-binding region.

ENZ_ACT	Enzymatic activity. Types of enzymatic reactions as a subpart to protein functions.	ACT_SITE	Amino acid(s) involved in the activity of an enzyme.

CELL	Cellular phenotype. Class denoting different cellular phenotypes that can be affected by structural or compositional changes of a protein.	N/A	

The association between both, a word *w *and a category *c*, was estimated based on their mutual information score

(1)

The association between the multi-word term  and a category *c *was computed by the sum of the associations of its words

(2)

where P*(*c*) is the probability of a category associated with a term. The categorisation of a multi-word term into one of the categories, amounts to the identification of the category c* that maximises the association *A*(*T*, *c*):

(3)

### Evaluation corpora

UniProtKb is a comprehensive protein knowledge base that contains manually curated functional annotations for proteins, their sequences, and their residues. It also contributes citation references (PMIDs) for relevant articles. However, the precise association of a citation and a protein residue in context of functional annotation is generally not available.

The test dataset for the developed functional annotation extraction is based on the citation references from UniProtKb. A Uniprot corpus was generated by retrieving abstract texts from MEDLINE, that are indexed by the knowledge base. From the 136,566 citations listed in UniProtKb, an almost complete set of 136,559 abstract texts was retrieved from MEDLINE. Although not all information presented in the UniProtKb are necessarily available in the Uniprot corpus, the Uniprot corpus is a starting point for the evaluation of the developed text mining modules. Two derived test corpora were generated from the Uniprot corpus: the gold standard corpus with manual annotation and the cross-validation corpus with automatically annotated information derived from UniProtKb. Table 4 summarises key features in both test corpora. The gold standard corpus (GC) was created through manual curation, since no suitable annotated corpora are available for this study. A random sample of 100 MEDLINE abstract texts was drawn from the Uniprot corpus, where every abstract text must contain the tri-occurrences of organism, protein and residue. Notice that the detection of the entities was based on the entity recognition systems described in the previous section, and therefore represents an approximation of true data selection.

**Table 4:** Test corpora for information extraction evaluation. Based on the citation references from UniProtKb a base corpus was generated by retrieving abstract texts from MEDLINE. Two test corpora were derived from this corpus: the gold standard corpus (GC), which resembles a manually annotated test set, and the cross-validation corpus (XC), which contains automatically assigned annotations based on information from UniProtKb.

Dataset	Gold standard corpus (GC)	Cross-validation corpus (XC1)	Cross-validation corpus (XC2)
Abstracts count	100	55,998	5,253
Method of annotation	manual	automatic	automatic
total/unique residues	362/262 (with 262/191 having residue name + residue sequence position)	N/A	N/A
total/unique proteins	990/511	N/A	N/A
total/unique organisms	323/123	N/A	N/A
total/unique associations	240/172 residue-protein-organism associations	NA/70,401 protein-organism as UTP	NA/68,008 protein-residue as URP
Application	Test the the type, amount and reliability of the extracted information (reproduction of manually annotated information).	Test set is assumed to contain the same type of information as GC, but certainty is not clear. Study the reproduction of information contained in the database.	Test set is assumed to contain the same type of information as GC, but certainty is not clear. Study the reproduction of information contained in the database.

From this set of 100 abstract texts, manual analysis results in four types of annotations. The first type is the annotation of the biological entities of organism, protein, and residue, while the second is the annotation of entity triplet associations, i.e. organism-protein-residue. In addition, text segments of sentences with a residue entity were annotated, if they represent keywords for functional annotation. Finally, the association of a keyword and a residue was also annotated in GC.

For the automatic evaluation of extracted data, a cross-validation corpus (XC) was derived from Uniprot corpus. This test set was used to analyse the performance of protein-organism (XC1) and residue-protein (XC2) associations. The test set was annotated automatically, i.e. the biological entities were detected with the same entity recognition systems. The documents in the Uniprot corpus were scanned for tri-occurrences of organism, protein, and residue in text and analysed, if the combinations of the four identifiers, (UID+TID+RID+PMID), can be found in the database. UID is the Uniprot ID, TID is the NCBI Taxonomy ID, RID is a residue identifier, which consists of a residue name + sequence position, and PMID is the PubMed identifier. If at least a single match was found, then the document was selected. For the non-matching combinations the corresponding annotations were removed from text. From the retained and validated associations of the identifiers, two sets of three identifier combinations were determined: UTP = (UID+TID+PMID), and URP = (UIP+RID+PMID). 70,401 UTPs from 55,998 abstract texts were determined for XC1, and correspondingly 68,008 URPs were derived from 5,253 MEDLINE articles in XC2.

### Evaluation methods

The performance of each process of the developed protein residue identification was scored against a manually annotated gold standard corpus. Proteins, where the protein entity recognition system and manual curation assigned the same entity were considered as true positives (TP). The same rule also applied for counting TP of residue and organism entity detections.

The evaluation of the entity triplet association detection considered only associations as TP, if both pair relations organism-protein and protein-residue were determined correctly. If one of the relations was incorrect, a found association was counted as false positive (FP).

In contrast, the automatic evaluations of the entity recognition and entity association detection systems were performed on XC. A true positive of an annotated entity within an abstract text was identified, if UniProtKb lists the same entity in context of the given PMID. For example, if organism X in text Y is also indexed in UniProtKb as a combination of TID+PMID, then a true positive was counted.

A correct protein-organism association was detected, if the determined identifier combination UTP was found in XC. Similarly, a correct residue-protein association was found, if the derived identifier combination URP was found in the test corpus. Notice that within this evaluation setup a UTP or URP was counted as FP if the information was not stored in UniProtKb albeit the correct entity association. The effectiveness of the entity recognition and the association detection systems was measured in terms of precision, recall and the balanced F-measure (F1).

The extraction of contextual features of residues results in a set of syntactical relations, which are represented as PAS. The performance of this extraction module was evaluated by comparing the returned PAS data with manual annotations in GC. A TP was counted, if the syntactical relations in a PAS were correct, and if the arguments in the PAS contained the annotated residue entity and the marked keyword(s) in the test corpus.

The performance of the developed classification method was evaluated by a 100 times 5-fold cross-validation. For each iteration, terms in the reference set were shuffled, and partitioned into a test set (1/5 of the data) and a training set (4/5 of the data). The average precision, recall and F1-measure were calculated for each classifier from the determined confusion matrix.

## Results

The following sections assess first the extraction system and then the extracted data.

### Evaluation of entity recognition: organism, protein, and residue entity

All presented results have been evaluated against the manually curated test set, i.e. the gold standard corpus (GC). Entity recognition for the residue mention yields to 92% precision and 98% recall (95% F1 measure). This performance is slightly higher than the one of previously reported solutions of point mutation identification [15,16,18], indicating the extended set of regular expressions are precise. The performance for protein mention identification is evaluated with 65% precision and 60% recall (62% F1 measure). The result is difficult to compare to previously reported systems, e.g. ProMiner and MutationMiner, due to the different experimental setup. ProMiner was evaluated on the BioCreAtIvE corpus (80% F1 measure) which links the contained protein mentions to only a small set of organisms. However, we have repeated the experiment on the BioCreAtIvE dataset and the result suggests that our method yields a comparable performance (76% F1 measure). Conversely, the evaluation of MutationMiner not only considers abstract texts but also the content of the full-text articles which should improve the results (79% F1 measure).

The performance of the organism entity recognition system shows better performance than the protein entity recognition solution (81% precision, 72% recall).

The identification of entity triplets consisting of one of each entity type, i.e. the protein, organism and residue mention (POR), is based on association rules. The identification resulted to 82% precision and 38% recall, where our precision is similar to other reported solutions. Our recall is lower than the one reported in MutationGraB and MuteXt and could be explained by the fact that both systems are focused on protein family specific full-text articles. According to our manual inspection the main cause for low recall is because the association of the protein to the organism is often not stated explicitly.

The POR detection still requires improvements but is sufficiently precise (82%) to use it for the annotation of protein residues with contextual features. Furthermore, the identification of the correct organism from the literature is helpful for the alignment of the finding with the correct database entry, but it is also an option to relax the organism identification and to rely more on the database content for the final resolution of the protein residue.

### Cross-validation of identified protein residues with UniProtKb

The POR triplets were now cross-validated against the content of UniProtKb which is the reference database for protein annotations using the cross-validation corpus (XC). We assessed the association of the protein to the organism independently from the residue-protein association. The comparison yielded 82% precision and a recall of 88% for the protein-organism. In the performance assessment of the residue-protein association detection we measured 83% precision and 13% recall. The low recall is less surprising in this case since the annotation of residues is not as mandatory as the species annotation in UniProtKb.

The cross-validation of both associations resulted to 54% F1 measure with 83% precision which is similar to our analysis against the GC. By contrast, the average recall in the cross-validation is higher than the one reported from analysis based on the gold standard. The reference database provides reliable information for comparison but the time-consuming curation process is one reason for the lack of content in comparison to the scientific literature.

**Figure 2:**
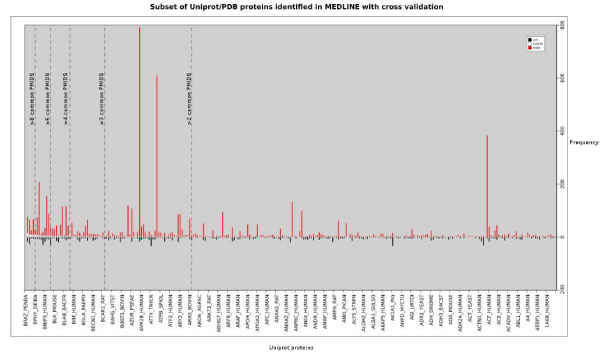
**Cross-validation of citations from identified protein residues with UniProtKb/PDB**. For a subset of identified UniProtKb/PDB proteins (i.e. proteins with UniprotID and PDBID) in MEDLINE, the determined PubMed identifiers (PMIDs) can be cross-validated with the relevant citation set from UniProtKb. uni = UniProtKb/PDB based citations; med = protein residue identification based citations; comm = common set of citations between uni and med.

Finally, we filtered all MEDLINE abstracts for mentions of POR triplets and compared the retrieved citations against the references in the citation sets in UniProtKb/PDB. In total 40,750 MEDLINE abstracts make a reference to POR triplets. In total 9,354 Uniprot proteins are covered and 2,884 of these proteins have hyperlinks to 14,007 PDB entries. These 2,884 shared protein mentions are linked to 18,427 MEDLINE abstracts, whereas UniProtKb/PDB makes only reference to 4,652 PMIDs out of which 657 PMIDs are shared between both resources. This demonstrates again that the MEDLINE abstracts do not necessarily cover the information that curators have deemed relevant (cf. figure 2). Taking into consideration 82% of the identified protein mentions are correct based on the GC evaluation the total number of relevant abstracts results to 15,110.

### Extraction of contextual feature for the annotation of protein residues

In the following we extracted the predicate-argument structure from the context of a protein mention based on shallow parsing and full parsing using ENJU. The shallow parser yielded 56% F1 measure (68% precision, 48% recall) and ENJU produced 31% F1 (37% precision, 27% recall). Manual inspection of the false positive results indicates that mistakes in the part-of-speech tagging are the main source of the error rate. For example, in the sentence

"Conversely, K382Q displays a highly altered responsiveness to the activator, suggesting that Lys(382) is involved in both activator binding and allosteric transition mechanism." (PMID:10751408),

both parsers identified "altered" as a verb in past tense, although the correct POS is a noun modifier. The performance of the POS tagger is critical for the detection of phrase boundaries.

Altogether, the developed shallow parser identifies predicate-argument structure from the context of residue entities. The extracted information is used as functional annotation.

### Extraction of functional annotations of protein residues from the context

The identification of functional annotations in the scientific literature is a challenging task. It requires the identification of textual features that can be linked to predefined classes. The categories of interest are listed in table 3 and have been compared to the feature set used in UniProtKb. The former categorization scheme is referred to as MAN and the latter as FEAT.

The classifiers have been trained on the proposed feature sets and the performance has been measured using a cross-validation scheme. For MAN, the best performing classifiers produced F1 measures of 62%, 57%, and 57% for the categories STR_COMP, CHEM_MOD and BINDING (see table 5). The micro-averaged F1-measure of 56% (see table 6)  is well above the calculated macro-averaged F1-measure of 43% indicating that the distribution of categories and examples is skewed. This again shows that the different classifiers vary in their performances to a significant extent. Similar results are produced for the performances of the classifiers that have been trained on the FEAT categorisation scheme.

**Table 5:** Performance evaluation of the classifiers (precision, recall, F1 measure). Classification with categories from MAN and FEAT were analysed by a 100 times 5-fold cross-validation.

MAN				FEAT			
	
Category	Precision	Recall	F1	Category	Precision	Recall	F1
STR_COMP	0.56	0.69	0.62	DOMAIN	0.50	0.24	0.32
				MOTIF	0.98	0.36	0.53
				TOPO_DOM	0	0	0
				CHAIN	0	0	0
				TRANSMEM	0	0	0
				COIL	0	0	0

CHEM_MOD	0.54	0.59	0.57	VARIANT	0.50	0.69	0.58
				MOD_RES	0.40	0.23	0.29
				PEPTIDE	0.05	0.06	0.05
				VAR_SEQ	0	0	0
				LIPID	1	0.32	0.48
				CARBOHYD	0	0	0

STR_MOD	0.24	0.10	0.15	REGION	0.44	0.44	0.44
				SITE	0.40	0.55	0.46

BINDING	0.63	0.52	0.57	BINDING	0.41	0.45	0.43
				METAL	0.05	0.02	0.03
				DISULFID	0.53	0.15	0.23
				CROSSLNK	0	0	0
				DNA_BIND	0	0	0
				NP_BIND	0	0.06	0
				ZN_FING	0	0	0
				CA_BIND	0	0	0

ENZ_ACT	0.43	0.20	0.27	ACT_SITE	0.45	0.31	0.36

CELL	0.50	0.31	0.38	N/A			

GEN_BIOL	0.70	0.64	0.67	GEN_BIOL	0.76	0.65	0.70

GEN_ENG	0.21	0.32	0.26	GEN_ENG	0.23	0.32	0.27

**Table 6:** Overall F1 measure of the entire classification. The global F1 measure of the classification problem is computed by two different types of averages: micro-average and macro-average. In micro-averaging, the F1 measure is calculated globally over all categories, while in macro-averaging, F1 is computed locally over each category first, and then the average over all categories is taken.

	MAN	FEAT
F1(micro-averaged)	0.56	0.55
F1(macro-averaged)	0.43	0.19

The confusion matrix (table 7) for the MAN categorization scheme reveals that the classifiers do not resolve properly the categories linked to general biological terms (GEN_BIOL) from the general English terms (GEN_ENG) showing that both categories share ambiguous terms. Other categories are not well separated either, e.g. STR_COMP against CHEM_MOD, and ENZ_ACT against STR_COMP, which demonstrates that the classes are not clearly disjoint. For example, "mutant structure" refers to an altered protein structure state, which is based on a chemical change in the protein sequence.

**Table 7:** Performance analysis of the classifiers (confusion matrix). Classification with categories from MAN were analysed by a 100 times 5-fold cross-validation. The result is represented as a confusion matrix.

Actual	Prediction							
	BINDING	GEN_BIOL	CELL	CHEM_MOD	GEN_ENG	ENZ_ACT	STR_COMP	STR_MOD

BINDING	**1,772**	762	28	93	165	26	546	0
GEN_BIOL	560	**15,815**	525	1,496	4,514	159	1,714	65
CELL	96	1,167	**836**	150	325	91	67	0
CHEM_MOD	38	1,103	12	**3,742**	761	79	546	25
GEN_ENG	144	2,556	126	510	**1,820**	46	480	35
ENZ_ACT	33	338	80	201	226	**324**	457	0
STR_COMP	160	783	64	551	592	35	**4,914**	11
STR_MOD	1	91	1	129	125	0	21	**43**

Due to the fact that the classifiers are applied to a large amount of data, we expect that the redundancy of the information in the scientific literature still allows us to identify sufficient textual features and to assign them to our predefined categories. For the future we expect that an increase in the number of training data and modifications to the size of the feature sets could improve the performance of our classifiers.

### Manual validation of extracted functional annotations and cross-validation with UniProtKb

The gold standard corpus consists of 100 abstract texts with tri-occurrences of the triplet protein, residue and organism. However, manual analysis identified only 51 abstract texts with residue entities that can be associated with their proteins and hosting organisms. The number of associations (POR) is 172. This represents the target for protein residue identification.

Corresponding to these PORs is the set of functional annotations (PAS data). For 109 out of 172 PORs, keywords were co-mentioned in verbal relations. The number of PAS associated with the 109 PORs is 117. This represents the target of functional annotation extraction.

**Figure 3:**
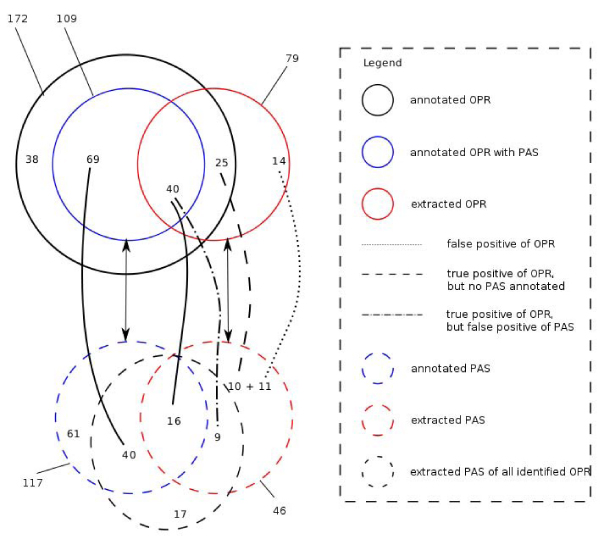
**Performance evaluation of the functional annotation extraction system**. Annotation extraction is dependent on the performances of two text mining modules: protein residue identification and contextual feature extraction. The analysis compares the extraction depending on both modules, and the extraction depending solely on contextual feature extraction, i.e. assuming all protein residues are correctly identified. The performance was measured in terms of precision, recall, and F1 measure.

Figure 3 summarises the performance of the functional annotation extraction. With a previously determined precision of 0.82 and a recall of 0.38, the protein residue identification module detects 79 PORs with 65 out of 79 being the correct ones. Contextual feature extraction for these 65 protein residues resulted in 35 PAS data. In comparison with the 117 annotated PAS of the 109 PORs, only 16 out of 35 extracted PAS are true positives. The total number of extracted PAS, however, is 46, which results in a precision of 0.35 and a recall of 0.13. A systematic analysis revealed, that the rate of false positives has the following sources: a false positive of POR with extracted PAS, a true positive POR with no annotated PAS, and a true positive of POR with false positive of PAS.

In comparison, if the system would have identified all protein residues correctly, the performance of the whole extraction would have yielded in a precision of 0.68 and a recall of 0.48. This shows that functional annotation can be extracted with a reasonable confidence. The recall can be explained by the performance of the contextual feature extraction module.

The result indicates that the extracted functional annotations have a reasonable precision, but are low in coverage. This can be explained by the sum of the performances of each text mining module. On one hand, an incorrectly determined protein residue leads to a false positive of PAS. On the other hand, failed entity recognition contributes to the false negative rate. In addition, language complexity, and incorrectly parsed sentences are the other reasons for the false positive and false negative rate of functional annotation extraction.

Despite the low coverage of the functional annotation extraction system, the extracted information is correct and reusable for the annotation of protein residues. A comparison with UniProtKb shows, that 5 out of 16 are rediscovered knowledge (cf. Additional file 2). The remaining 11 out of 16 contain novel information that can be used to update the protein knowledge base.

In conclusion, the presented functional annotation extraction system delivers precise information, but has a low coverage of extraction. Although the performances of each module may not be at optimal level, the results demonstrate that functional annotations can be extracted from MEDLINE.

## Discussion

The presented text mining solution extracts textual features from the context of residue entities. Although the identification of a residue-keyword association can be attempted with co-occurrence analysis, the target is to extract reliable associations with contextual information on their association. We analysed the syntactical structure of a sentence and extract verbal and prepositional relations of noun phrases, where one relation element contains a protein residue. The advantage is not only to find explicitly stated associations between residue and keywords, but also the relation type and context of association.

The extracted information is difficult to normalise, because there is no gold standard of how to represent the association, and how to qualify the contextual information. We adopted the idea of using predicate-argument structure as a template for the extracted information. Although verb frame sets from PropBank or PASBio can be used to normalise the extracted data, they are not designed to capture protein residue function. Conversely, this gives the developed extraction approach the advantage to discover new knowledge for annotation.

To identify descriptions of protein function in text, terminologies from Gene Ontology can be used. However, this ontology is currently not optimized for protein residue annotation, for example the term "active site" does not even appear as a stand-alone term in the data resource (cf. table 8 for further examples). Generally, description of protein function refers to a higher level of biological function, e.g. metabolomics or cell signalling. In contrast, the annotation of protein residues requires a different set of terminologies that describe molecular interactions or chemical reactions. At the moment, an ontology dedicated solely for the functional annotation of protein residues has not been developed, and terminologies have to be collected from various resources.

**Table 8:** GO terms are not suitable for protein residue annotation. The presented examples demonstrate that predicted GO terms are not always suitable for protein residue annotation. The prediction of GO terms was done with an information theory based parser [34].

		Annotation	
		
Example	Sentence	Manual	GO
1	"The catalytic mechanism of the non-phosphorylating glyceraldehyde-3-phosphate dehydrogenase and the other aldehyde dehydrogenases resembles a thioester mechanism involving the universally conserved cysteine 298 (pea GAPN)." (PMID:9461340)	thioester mechanism, conserved cysteine	glyceraldehyde-3-phosphate dehydrogenase (NADP+)(phosphorylating activity), glyceraldehyde-3-phosphate biosynthesis, glyceraldehyde-3-phosphate catabolism, phosphoglycerate dehydrogenase activity

2	"However, mutations of a key residue, His48, show significant deviation from the relationship, implying a role for the side chain in protection of the complex from hydroxide attack." (PMID:2690955)	protection of the complex from hydroxide attack	AT DNA binding, tRNA, tyrosine tRNA ligase activity

3	"Second, this reactive cysteinyl residue, which is required for L-cysteine desulfurization activity, was identified as Cys325 by the specific alkylation of that residue and by site-directed mutagenesis experiments." (PMID:81615929)	L-cysteine desulfurization activity	pyridoxal biosynthesis, phosphate binding, mutagenesis, nitrogenase activity, L-alanine biosynthesis, pyridoxal phosphate binding

The evaluation of the classification method indicates that the presented approach can provide an automatic solution for text interpretation. However, some of the categories have only few examples, which lower the performance of the classifiers. A solution to this problem is to balance the example sets of each category, for example, by collecting more terminologies from MEDLINE.

Despite the fact, that semantic labels can be assigned to the arguments in a PAS, the developed method is not able to interpret the meaning of the whole extracted text segment. For example, in the sentence

"Specific binding of the WT and mutant receptors Cys14Ala and Cys199Ala was inhibited in the presence of the disulfide bond reducing agent, DTT, implying that disulfide bonds are formed and can be reduced in these mutant receptors." (PMID:9202220).

The following information was extracted and semantic categories were assigned to the arguments of the PAS

pred = inhibited

arg1 = Specific binding

arg1-of = [the WT and mutant receptors CYS14ALA and CYS199ALA]/CHEM_MOD

arg2-in = the presence

arg2-of = the disulfide bond reducing agent.

Although, one part of the information in the example has been correctly assigned with the label CHEM_MOD, the entire text phrase should be labeled with BINDING. A solution to this problem is not trivial and requires several levels of linguistic analysis.

## Conclusion

The aim of this work was to compile protein residue features from MEDLINE texts as annotation for UniProtKb proteins by combining a series of previously studied text mining methods. Although the performances of each module may not be at optimal level, the generated data output indicates that the strategy is able to deliver biological meaningful results. Cross-validation with UniProtKb analysis indicates that the extraction contains novel information that can complement and update the knowledge in UniProtKb and consequently provide annotations for PDB protein structures. However, as with high performing biological entity recognition and relation extraction systems become more available, this conceptual strategy in protein residue annotation extraction may yield optimal results for the biological community.

It is important to note that the extraction was done only on abstract texts from MEDLINE. The benefits lie in the broad coverage of biological topics, and the open access to a vast amount of scientific articles, which are advantageous for evaluating the biological significance of residues in proteins from various biological context. However, we acknowledge that the goal in this specialised information extraction task is to be able to find relevant data from full text articles. Considering that full text documents contain more information than abstract texts, and that this setup is more advantageous for previously reported point mutation identification systems, it is interesting to see how the proposed functional annotation extraction system performs on full text articles.

## Competing interests

The authors declare that they have no competing interests.

## Authors' contributions

Kevin Nagel carried out the experiments, developed and implemented the methods, assessed the annotations, and drafted the manuscript. Antonio Jimeno-Yepes participated in the development of the methods and drafted the manuscript. Dietrich Rebholz-Schuhmann participated in design of the experiments, assessed the annotation and drafted the manuscript. All authors read and approved the final manuscript.

## Supplementary Material

Additional file 1 **Labeled termlist according to the MAN scheme**. The listed terms were found according to a substring analysis of noun phrases with identified protein residues with the whole string of other coocurring noun phrases (MLNP analysis). The terms were manually assigned to categories from the categorisation scheme MAN. Click here for file

Additional file 2 **Comparison of extracted functional annotations from GC with UniProtKb**. Comparison of extracted protein residue annotations from GC with UniProtKb. Mined functional annotations are listed as PAS, while relevant information from UniProtKb are reproduced from the feature table entry line. Click here for file
